# Supplementary material for: PCH1 and PCHL promote photomorphogenesis in plants by controlling phytochrome B dark reversion
Source: Nat Commun. 2017 Dec 20;8:2221. doi: 10.1038/s41467-017-02311-8 (PMC5738371; doi:10.1038/s41467-017-02311-8)
Supplement: Supplementary file 1 — Supplementary Information [file 41467_2017_2311_MOESM1_ESM.pdf]

SUPPLEMENTARY FIGURES

a

Peptide counts dark	Peptide counts red	Unique peptides	Protein coverage	Accession	Protein
2615	3615	108	0.710751	AT2G18790	phyB
1	11	8	0.160935	AT2G16365	PCH1

```
>sp|Q84V03-2|FB348_ARATH Isoform 2 of F-box protein At2g16365
OS=Arabidopsis thaliana GN=At2g16365
MSEHVMVLGKGNKGISKNSSVNPYESAWLGRWTQSGSEVKFHDGETNCSKQLIRPEDENH
GVEVLPFPMFKVSQKRETTTTTTTKPSFHEDVGSSSRAMVNRMPWMYPQGENFSSSNRLD
FPIQEKTTQNLLELIRPVRIYATVDSVNLPKEDSHQLLKGSTVSMKLKGKIFGGYLDLFP
NQDHSHNRGGVRLQSLESSKDTQEDGPRKNESSAETNTLEMDRLQTIHLSGSISSSSTKG
KGIKGYSAIPRTEIPDMNEEPPLVPDRENSVDGHQGETSNSATQSMNVEHFLSRDCKRVR
LEPEVEASSRWVKRLKTSPSDSSETKSMMKMKEASLGEKENNNLFLEILKSGINNLQPRN
QEPVVSQSNDLRQGGDDITLLHPWIQRWCKKKTTSTDQPTGQEASFEPESHKEFEKKQYP
SIAAMALMGKALSGLNPYGLRKTNSLMVWNARDLR
```

b

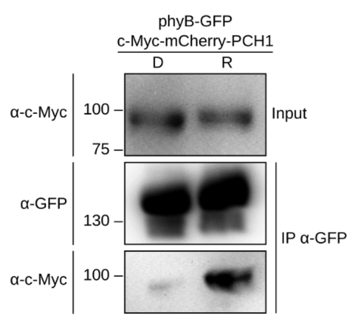

c **d**

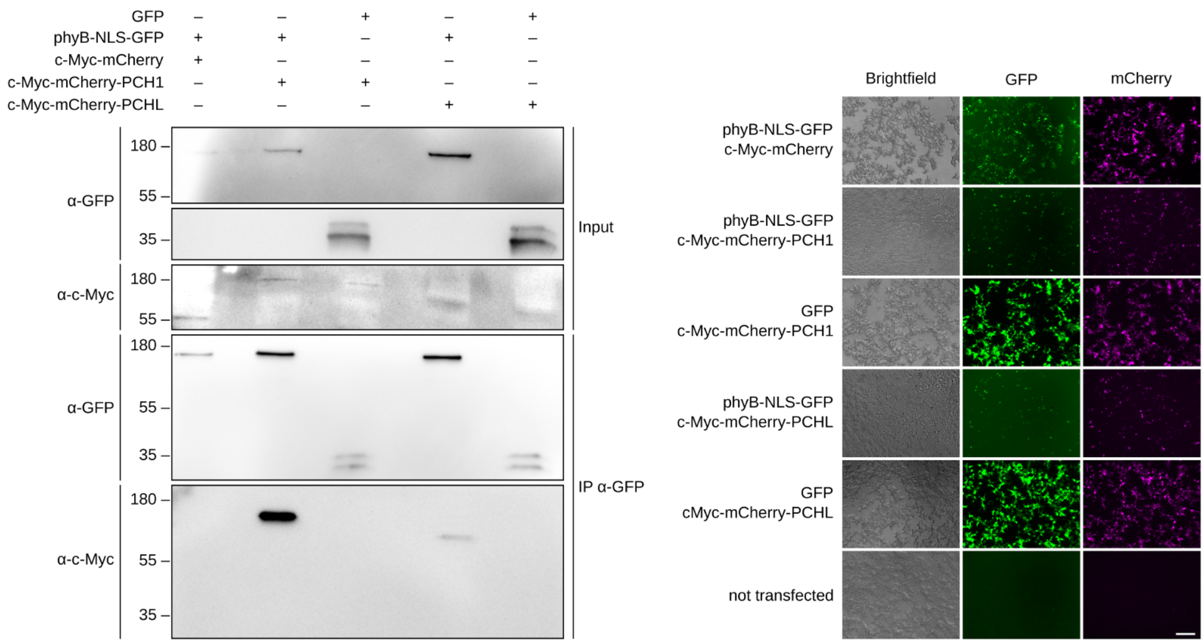

Supplementary Figure 1. PCH1 and PCHL interact with phyB. a MS identification of PCH1 as a phyB interacting protein. phyB-NLS-GFP was immunoprecipitated from seedlings grown in the dark and exposed to red light (7 µmol m^{-2} s^{-1}) for 10 min; control seedlings were kept in darkness. Proteins co-immunoprecipitating with phyB were analysed by MS/MS. Peptide counts are shown in the table. Individual peptides mapping to PCH1 are shown below (in different colours and underlined). **b** Co-immunoprecipitation of phyB-GFP and c-Myc-mCherry-PCH1 from stable transgenic *Arabidopsis*. Four day-old dark-grown seedlings co-expressing c-Myc-mCherry-PCH1 and phyB-GFP were either treated with red light (R, 7 µmol m^{-2} s^{-1}) for 10 min or kept in darkness (D). Total native protein extracts were prepared and immunoprecipitation performed using α-GFP antibody. α-c-Myc and α-GFP antibodies were used to detect c-Myc-mCherry-PCH1 and phyB-GFP, respectively. **c** Co-immunoprecipitation of phyB and PCH1/PCHL from mammalian cells. PhyB was expressed as a nuclear-localisation-signal (NLS)-GFP fusion in HEK293T cells with either c-Myc-mCherry-tagged PCH1 or PCHL. Immunoprecipitations were performed using total native protein extracts and α-GFP labelled beads. α-GFP and α-c-Myc antibodies were used to detect phyB, PCH1, and PCHL in both input and elution. PhyB in HEK293T cell extracts was not photoactive under the conditions used, preventing analysis of the effects of light on the

interaction. Levels of PCH1 and PCHL in HEK293T cells are too low to reliably detect by immunoblotting. **b, c** The molecular size marker (kDa) is shown on the left. **d** Expression of PCH1 and PCHL in HEK293T cells can be detected by fluorescence microscopy. GFP and mCherry specific filter sets were used to visualise expression of phyB-NLS-GFP and c-Myc-mCherry-PCH1/-PCHL in HEK293T cells used for Co-IP assays in **c**. Scale bar = 100 µm.

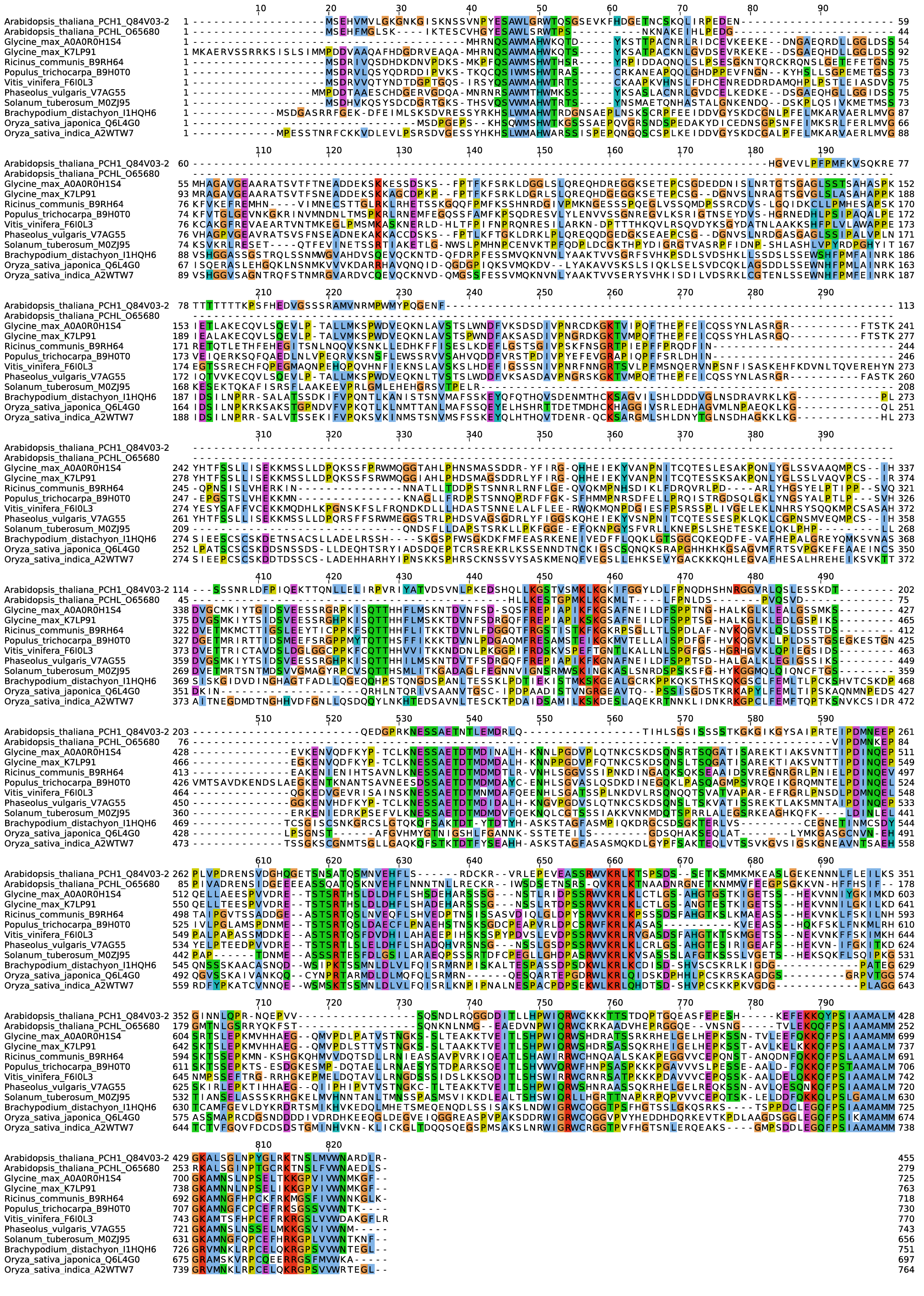

Supplementary Figure 2. Sequence alignment for PCH1/PCHL related proteins. *Arabidopsis* PCH1 and PCHL were used for a BLAST search at www.uniprot.org. PCH1/PCHL related sequences from different species were aligned to *Arabidopsis* PCH1 and PCHL using CLUSTALW [1,2] with default settings. Sequences are named by the species name and their UniProt identifier. The upper two sequences are *Arabidopsis* PCH1 and PCHL.

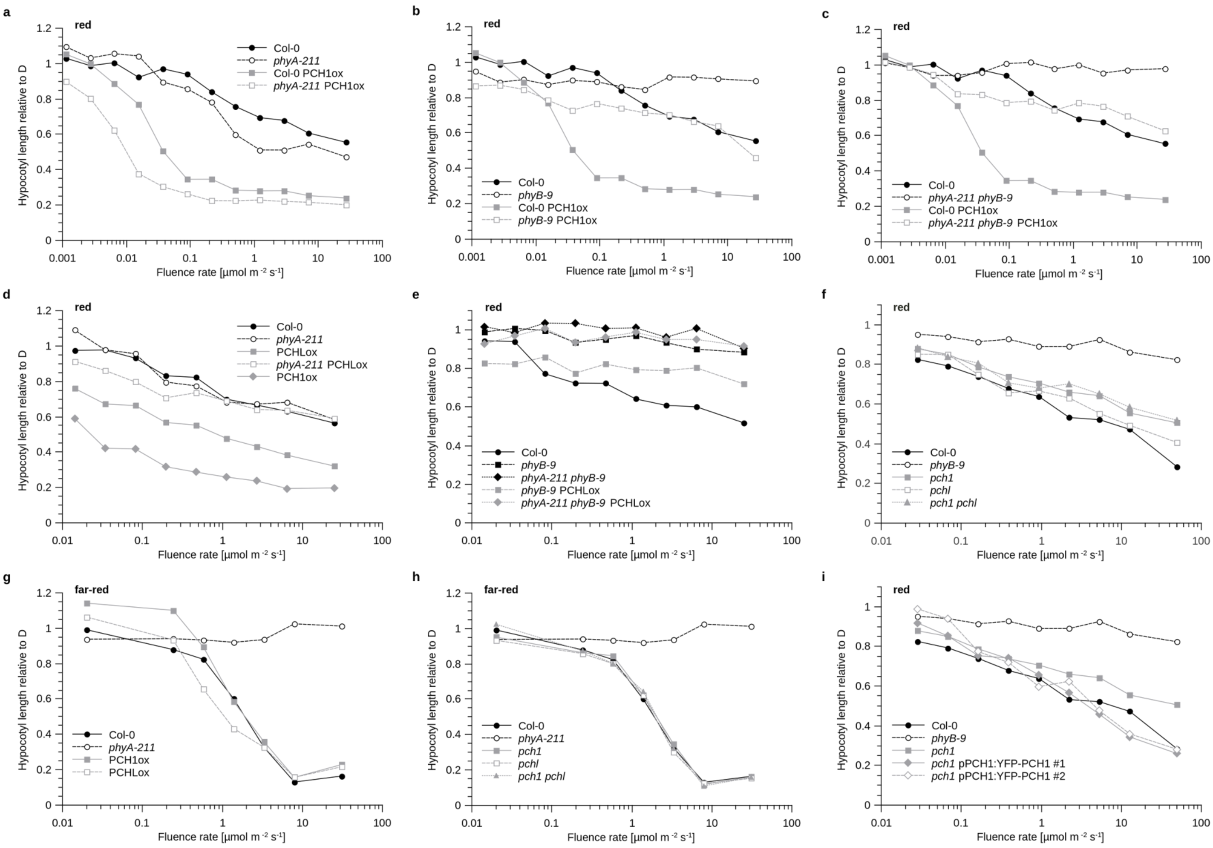

Supplementary Figure 3. PCH1 and PCHL promote photomorphogenesis under continuous monochromatic red light. a–c The red light hypersensitivity of PCH1ox seedlings strongly depends on phyB. Wild type (Col-0), *phyA-211*, *phyB-9*, *phyA-211 phyB-9*, as well as seedlings over-expressing HA-YFP-PCH1 (PCH1ox) in wild type, *phyA-211*, *phyB-9*, or *phyA-211 phyB-9* backgrounds were grown for 4 days in red light of different fluence rates. Data for Col-0 and PCH1ox in **a–c** are identical. **d, e** PCHLox seedlings are hypersensitive to red light. Wild type (Col-0), *phyA-211*, *phyB-9*, *phyA-211 phyB-9*, and seedlings over-expressing HA-YFP-PCHL (PCHLox) in wild type or mutant backgrounds were grown in red light of different fluence rates. Col-0 PCH1ox seedlings were included for comparison. **f** The *pch1* mutant is hyposensitive to red light. Wild type (Col-0), *phyB-9*, *pch1*, *pchl*, and *pch1 pchl* seedlings were grown for 4 days in red light of different fluence rates. Data for Col-0, *phyB-9*, and *pch1* in **f** and **i** are identical. **g** Over-expression of PCH1 and PCHL only slightly affects hypocotyl growth in far-red light. Wild type (Col-0), *phyA-211*, PCH1ox, and PCHLox seedlings were grown for 4 days in far-red light of different fluence rates. Data for Col-0 and *phyA-211* in **g** and **h** are identical. **h** The *pch1* mutant normally responds to far-red light. Wild type (Col-0), *phyA-211*, *pch1*, *pchl*, and *pch1 pchl* seedlings were grown for 4 days in far-red light of different fluence rates. **i** Complementation of the *pch1* mutant. Wild type (Col-0), *phyB-9*, *pch1*, and *pch1* expressing HA-YFP-PCH1 under the control of the *PCH1* promoter (#1 and #2, two independent transgenic lines) were grown for 4 days in red light of different fluence rates. **a–i** Data show mean hypoctoyl length relative to dark-grown seedlings. At least 20 seedlings were measured per line and treatment. Error bars are not indicated for clarity; ±s.e.m. is less than 0.04 for all lines/treatments.

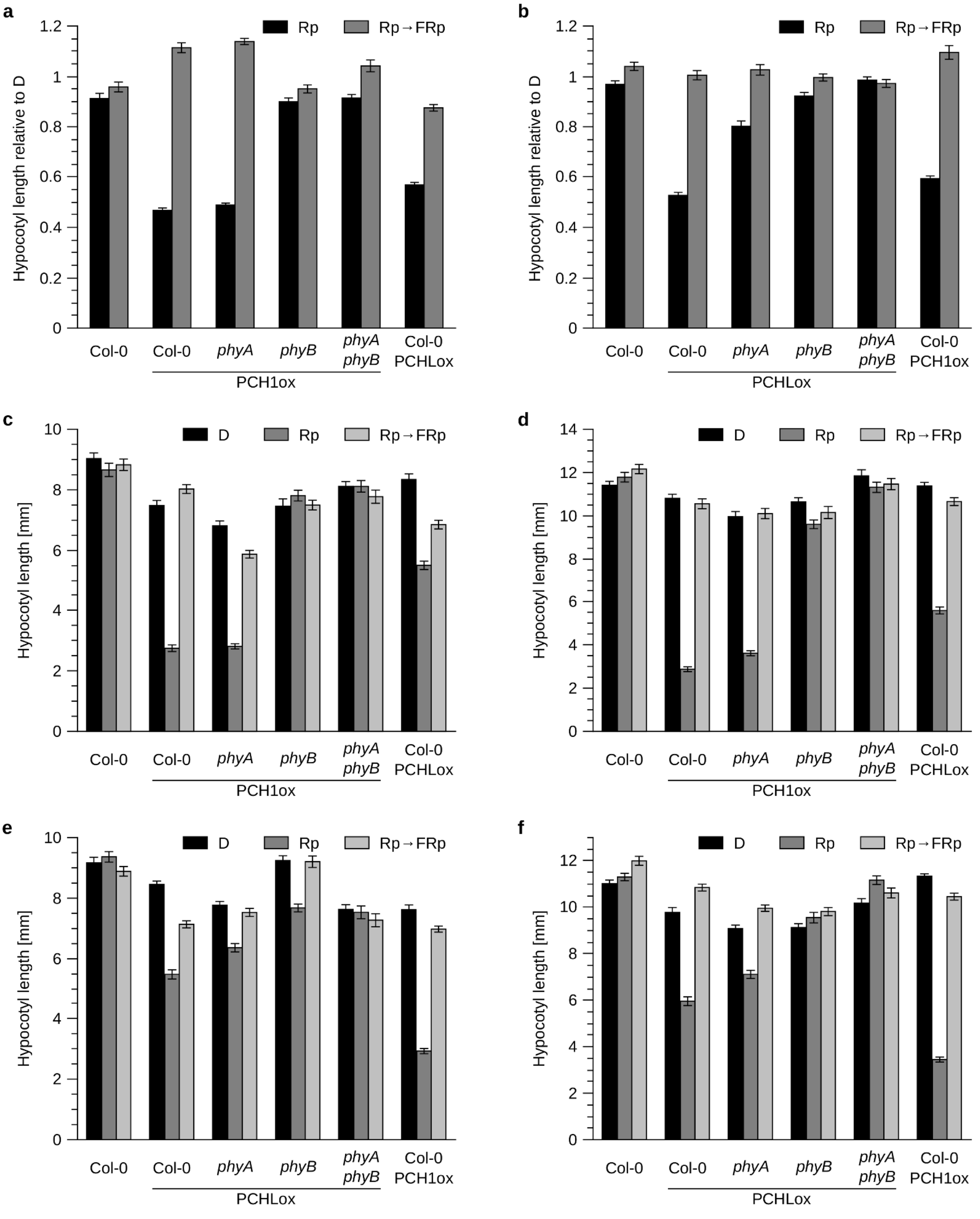

Supplementary Figure 4. PCH1ox and PCHLox seedlings respond to red light pulses in a phyB-dependent manner. a, b Quantification of Fig. 2a and 2b. Seedlings over-expressing HA-YFP-PCH1 (PCH1ox) (**a**) or HA-YFP-PCHL (PCHLox) (**b**) in different genetic backgrounds (Col-0, *phyA-211*, *phyB-9*, *phyA-211 phyB-9*) were grown for 4 days in darkness on filter paper soaked with water. The seedlings were either treated with a single red light pulse (Rp, 5 min, 50 µmol m^{-2} s^{-1}) per day, or a Rp followed by a long-wavelength FR pulse (FRp, 776 nm, 5 min, 50 µmol m^{-2} s^{-1}) (Rp→FRp). Control seedlings were not exposed to light. Mean hypocotyl length relative to dark-grown seedlings (±s.e.m.; $n \geq 20$) is shown. **c–f** The response to red light pulses is similar in seedlings grown on water and on 0.5× MS

medium. Seedlings over-expressing HA-YFP-PCH1 (PCH1ox) (**c, d**) or HA-YFP-PCHL (PCHLox) (**e, f**) in different genetic backgrounds were grown on filter paper soaked with water (**c, e**) or 0.5× MS medium (**d, f**) under the conditions described in **a** and **b**. Mean hypocotyl length of seedlings (±s.e.m.; n ≥ 20) is shown.

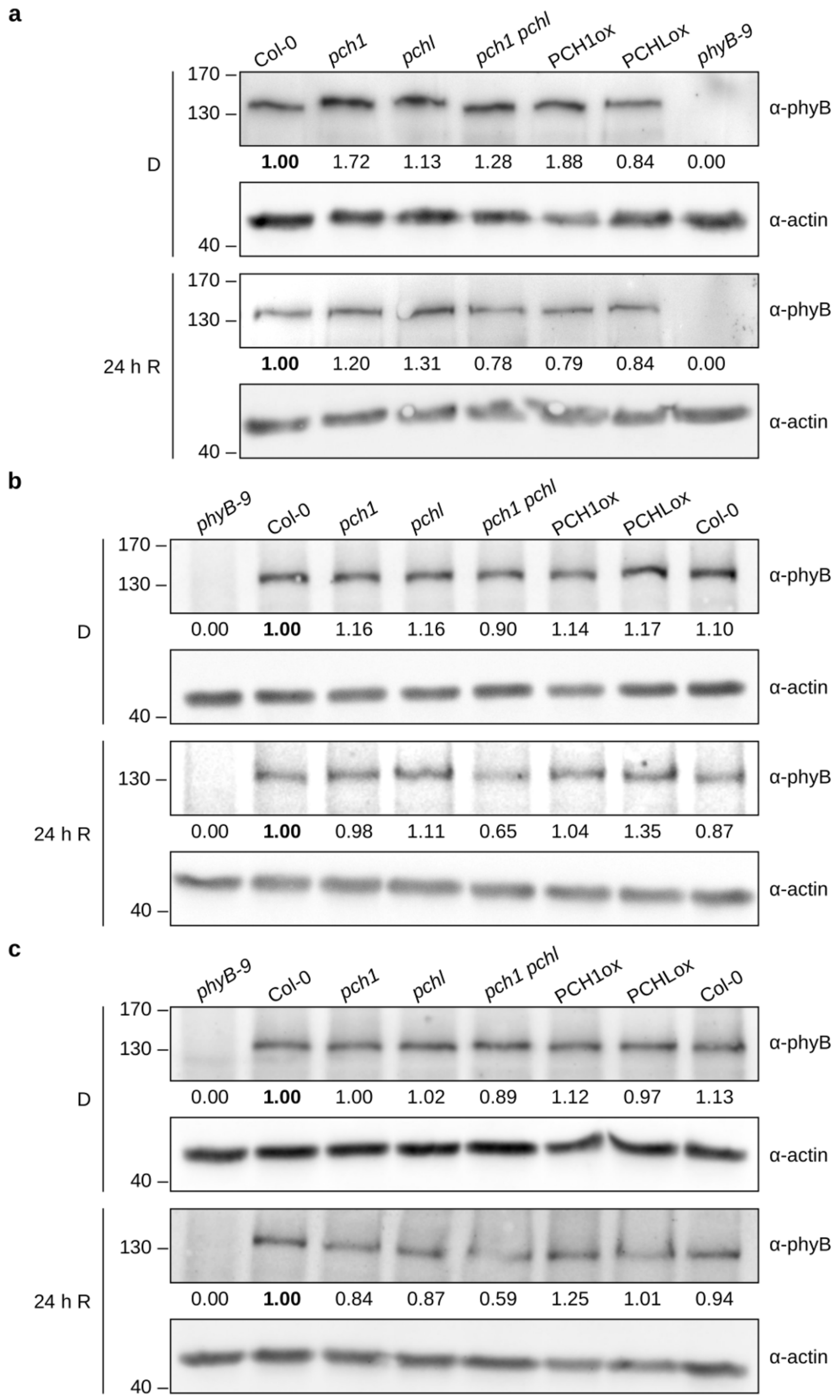

Supplementary Figure 5. PCH1 and PCHL do not alter phyB protein levels. a–c Three day-old etiolated wild type (Col-0), *pch1*, *pchl*, *pch1 pchl*, PCH1ox (p35S:HA-YFP-PCH1), PCHLox (p35S:HA-YFP-PCHL), and *phyB-9* seedlings were exposed to red light (R, 3 µmol m^{-2} s^{-1}) for 24 h or kept in darkness (D). Total protein was extracted and analysed by immunoblotting. α-phyB and α-actin antibodies were used to detect endogenous phyB, and actin as loading control. The molecular size marker (kDa) is shown on the left. **a–c** show three independent experiments with independent protein extracts. Bands have been quantified in each experiment; numbers indicate relative signal strength compared to Col-0 (shown in bold). In **b** and **c** the Col-0 protein extract was loaded twice to assess the reliability of quantification.

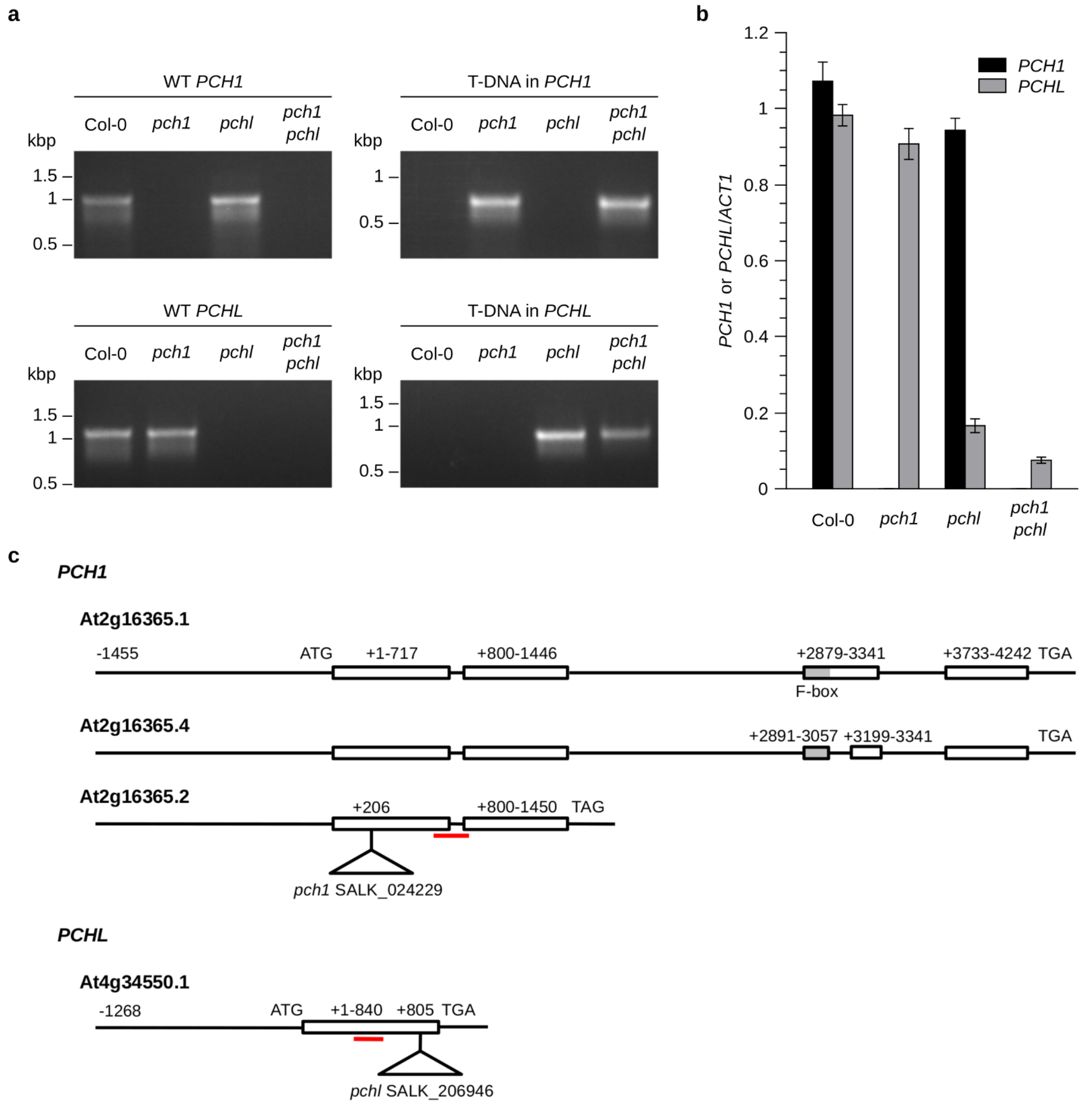

Supplementary Figure 6. Genotyping of *pch1* and *pchl* T-DNA insertion lines. a T-DNA insertion in *pch1* and *pchl*. Genomic DNA was isolated from wild type (Col-0), *pch1* (SALK_024229), *pchl* (SALK_206946), and *pch1 pchl* seedlings. Primers specifically detecting wild type *PCH1* or *PCHL* or the T-DNA insertion in the respective genes were used for genotyping by PCR. The ethidium bromide stained agarose gel is shown. **b** *PCH1* and *PCHL* transcript levels are strongly reduced in *pch1 pchl*. Wild type (Col-0), *pch1*, *pchl*, and *pch1 pchl* seedlings were grown for 4 days in the dark on filter paper soaked with water. 24 h prior to harvesting they were transferred to far-red light (740 nm, 25 µmol m^{-2} s^{-1}) to induce expression of *PCH1* and *PCHL*. Total RNA was extracted and qRT-PCR was performed using probes specific for either *PCH1* or *PCHL*. *ACT1* was used as internal control and expression of *PCH1* and *PCHL* is shown relative to expression of *ACT1*. Data are means of three technical replicates; error bars indicate ±s.d. **c** Gene models for *PCH1* (At2g16365) and *PCHL* (At4g34550). At2g16365.1 and At2g16365.4 encode a predicted F-box domain, indicated in grey, which is not present in the isoform At2g16365.2 used in this and a previous study [3]. At4g34550 does not encode a predicted F-box domain. The sites for the T-DNA insertions in *pch1* and *pchl* are indicated. Regions targeted for qRT-PCR analysis are indicated by a red line.

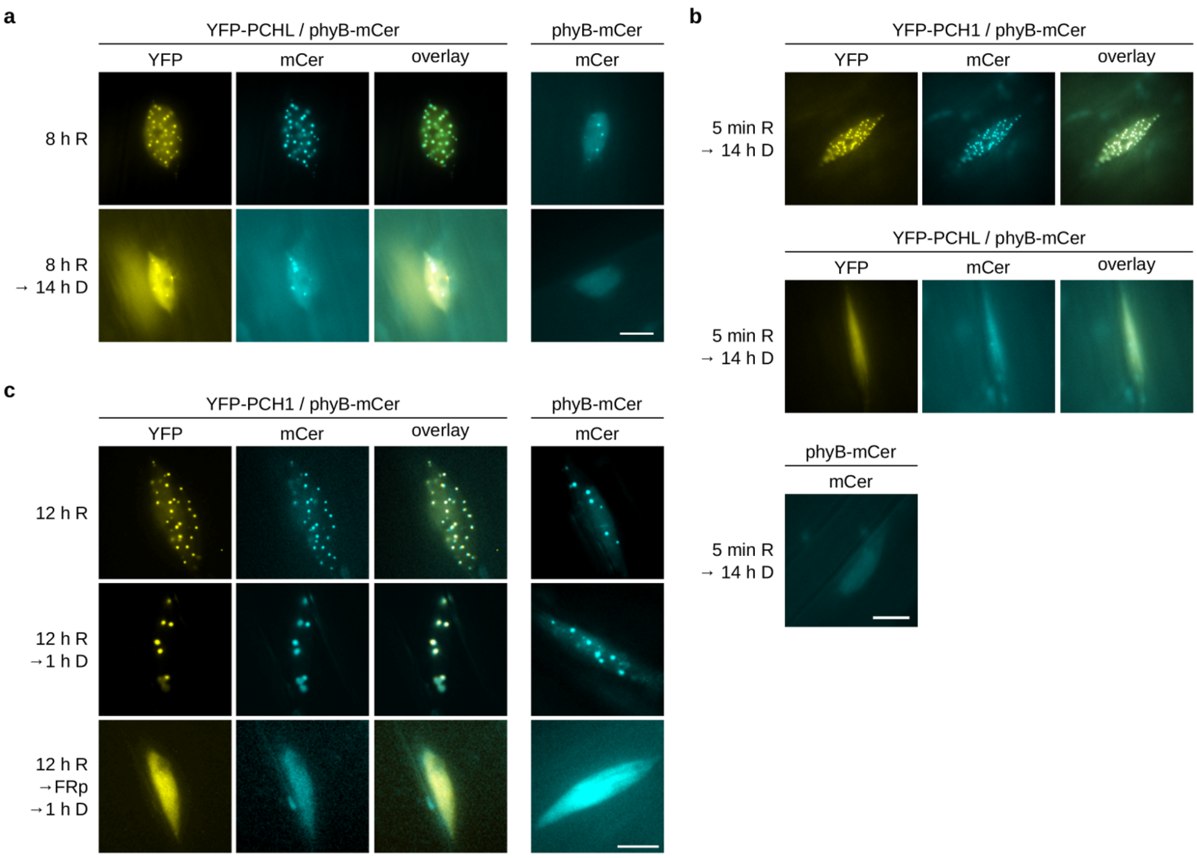

Supplementary Figure 7. PCH1 and PCHL maintain high levels of active phyB during the dark phase. a PCHL stabilises phyB photobodies. Four day-old etiolated seedlings expressing phyB-mCer in *phyB-9* or HA-YFP-PCHL (PCHLox) backgrounds were exposed to red light (R, 50 µmol m^{-2} s^{-1}) for 8 h or further incubated for 14 h in darkness (D) following the light treatment. Subnuclear localisation of HA-YFP-PCHL and phyB-mCer was analysed by fluorescence microscopy. Scale bar = 5 µm. Data for phyB-mCer single transgenic seedlings are duplicated from Fig. 2e. **b** PhyB photobodies induced by a red light pulse are stable in the dark in PCH1ox seedlings. Four day-old etiolated seedlings expressing phyB-mCer in *phyB-9*, HA-YFP-PCH1 (PCH1ox), or HA-YFP-PCHL (PCHLox) backgrounds were exposed to red light (R, 50 µmol m^{-2} s^{-1}) for 5 min, and further incubated for 14 h in darkness (D). Subnuclear localisation of HA-YFP-PCH1 and -PCHL and phyB-mCer was analysed by fluorescence microscopy. Scale bar = 5 µm. **c** Far-red light induced dissociation of phyB photobodies is normal in PCH1ox seedlings. Four day-old etiolated seedlings expressing phyB-mCer in *phyB-9* or HA-YFP-PCH1 (PCH1ox) backgrounds were exposed to red light (R, 20 µmol m^{-2} s^{-1}) for 12 h, or further incubated for 1 h in darkness (D) following the light treatment with or without a reverting long-wavelength far-red light pulse (FRp, 776 nm, 5 min, 50 µmol m^{-2} s^{-1}). Subnuclear localisation of HA-YFP-PCH1 and phyB-mCer was analysed by fluorescence microscopy. Scale bar = 5 µm.

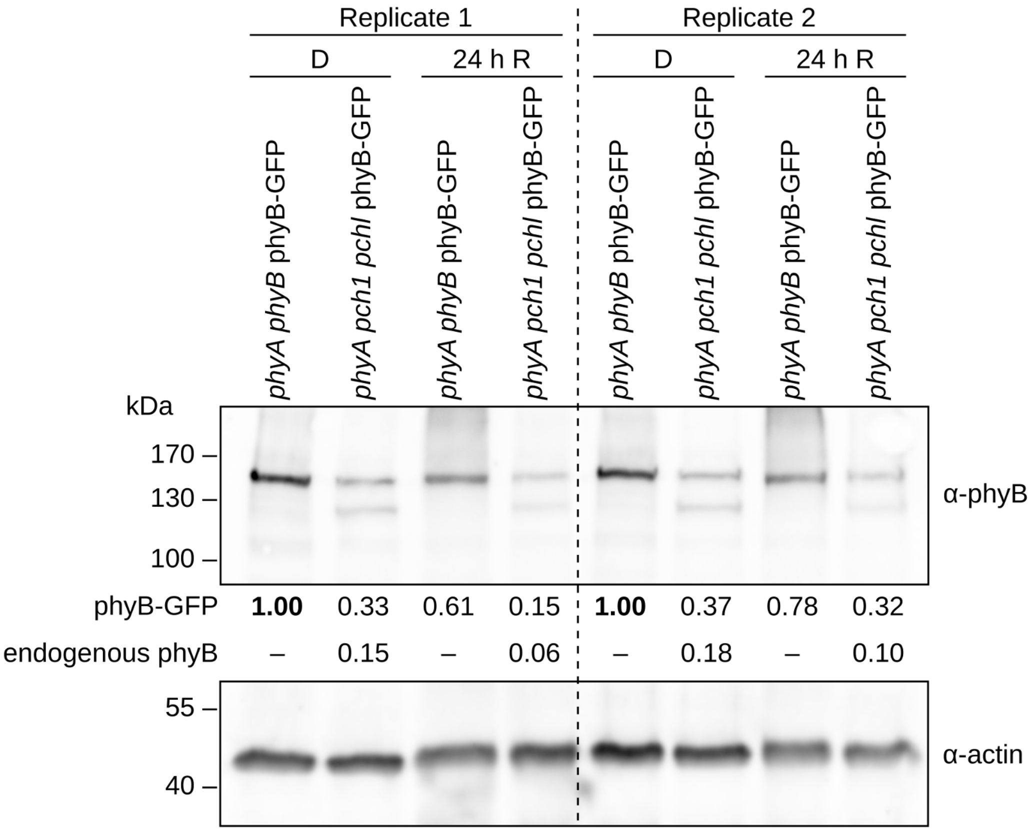

Supplementary Figure 8. Western blot quantification of phyB-GFP and endogenous phyB in lines used for *in vivo* measurement of Pfr. Four day-old etiolated seedlings expressing phyB-GFP in *PCH1 PCHL* wild type (*phyA phyB* phyB-GFP) or *pch1 pchl* mutant background (*phyA pch1 pchl* phyB-GFP) were exposed to red light (R, 3 µmol m^{-2} s^{-1}) for 24 h or kept in darkness (D). Total protein was extracted and analysed by immunoblotting. α-phyB and α-actin antibodies were used to detect endogenous phyB, and actin as loading control. Replicate 1 and 2 are experiments with independent protein extracts. Bands have been quantified for each replicate; numbers indicate relative signal strength compared to dark-grown *phyA phyB* phyB-GFP of the respective replicate (shown in bold).

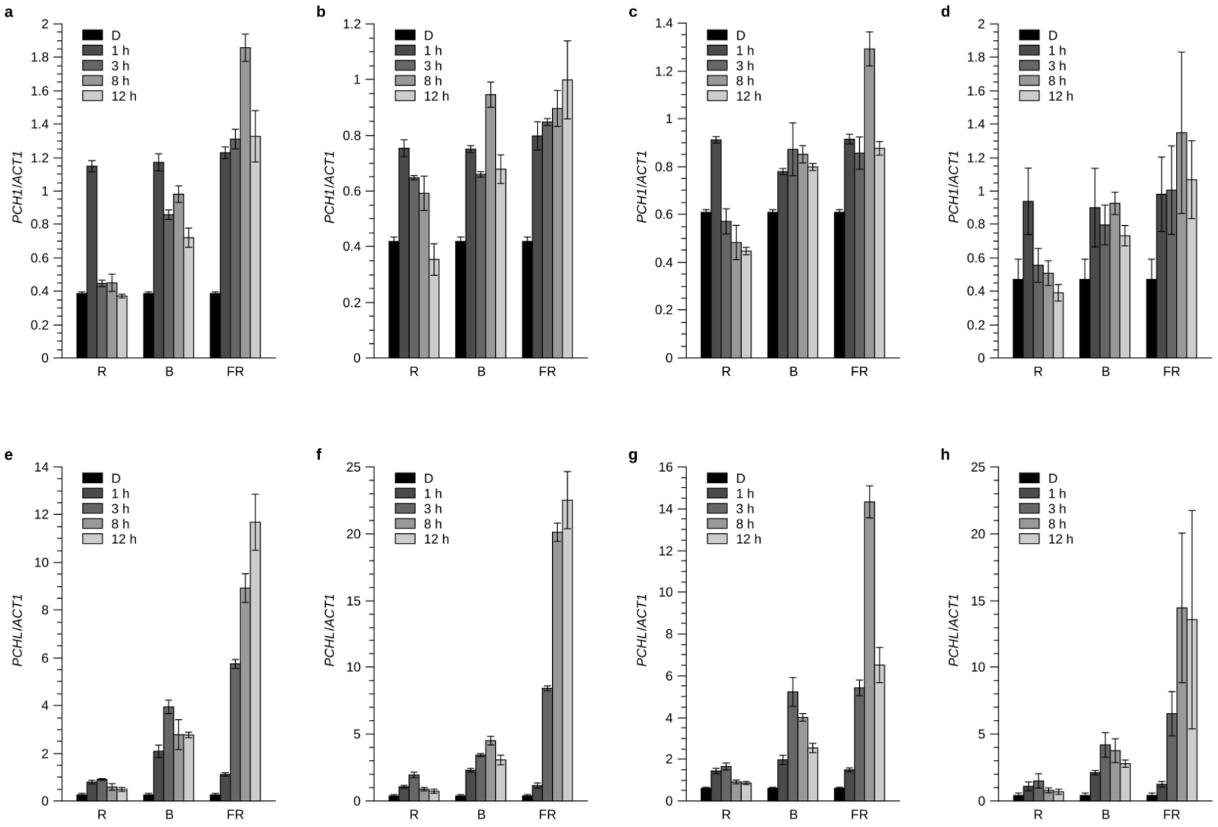

Supplementary Figure 9. Upregulation of *PCH1* and *PCHL* expression by monochromatic lights. a–h Four day-old dark-grown wild type seedlings were either kept in darkness (D) or exposed to red (R, 7.5 µmol m^{-2} s^{-1}), blue (B, 5.5 µmol m^{-2} s^{-1}), or far-red light (FR, 6.7 µmol m^{-2} s^{-1}) for the time indicated. Total RNA was extracted and qRT-PCR was performed using probes specific for either *PCH1* or *PCHL*. *ACT1* was used as internal control and expression of *PCH1* and *PCHL* is shown relative to expression of *ACT1*. Three biological replicates are shown for *PCH1* (**a–c**) and *PCHL* (**e–g**). Data of each biological replicate are means (±s.d.) of three technical replicates. **d, h** Bars show means (±s.d.) of the biological replicates.

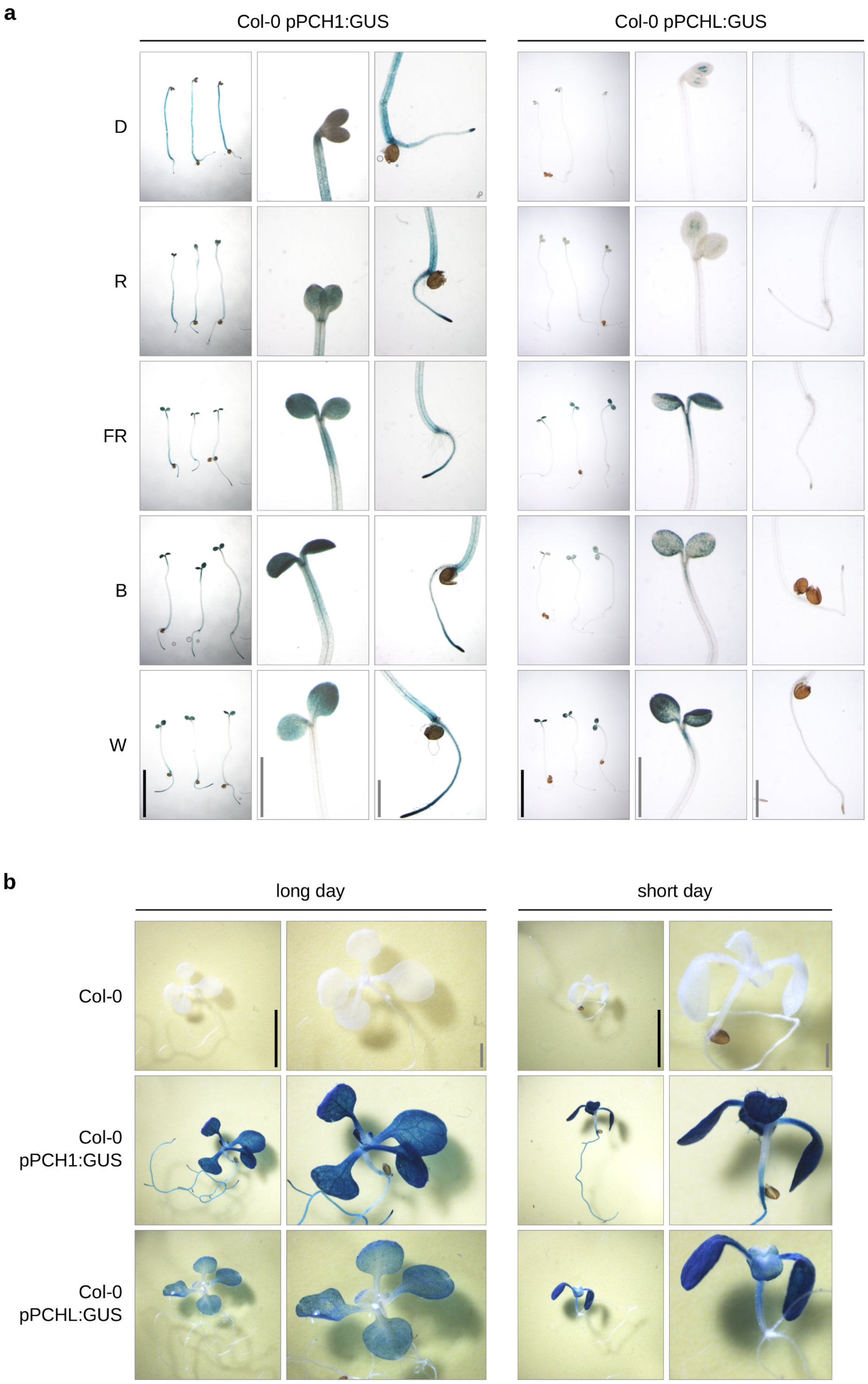

Supplementary Figure 10. Spatio-temporal analysis of *PCH1* and *PCHL* promoter activity. a Three day-old etiolated Col-0 pPCH1:GUS or Col-0 pPCHL:GUS seedlings were exposed to red (R, 25 µmol m^{-2} s^{-1}), far-red (FR, 25 µmol m^{-2} s^{-1}), blue (B, 25 µmol m^{-2} s^{-1}), or white light (W, 100 µmol m^{-2} s^{-1}) for 24 h or kept in the dark and then stained for GUS activity. Apical hook and cotyledons in dark-grown seedlings were closed when the seedlings were harvested and opened due to the staining/destaining procedure. **b** Col-0, Col-0 pPCH1:GUS, and Col-0 pPCHL:GUS plants were grown for 14 days under either long day (16 h W, 100 µmol m^{-2} s^{-1}/8 h D) or short day conditions (8 h W, 100 µmol m^{-2} s^{-1} /16 h D), and then stained for GUS activity. **a, b** Black scale bars = 5 mm; grey scale bars = 1 mm.

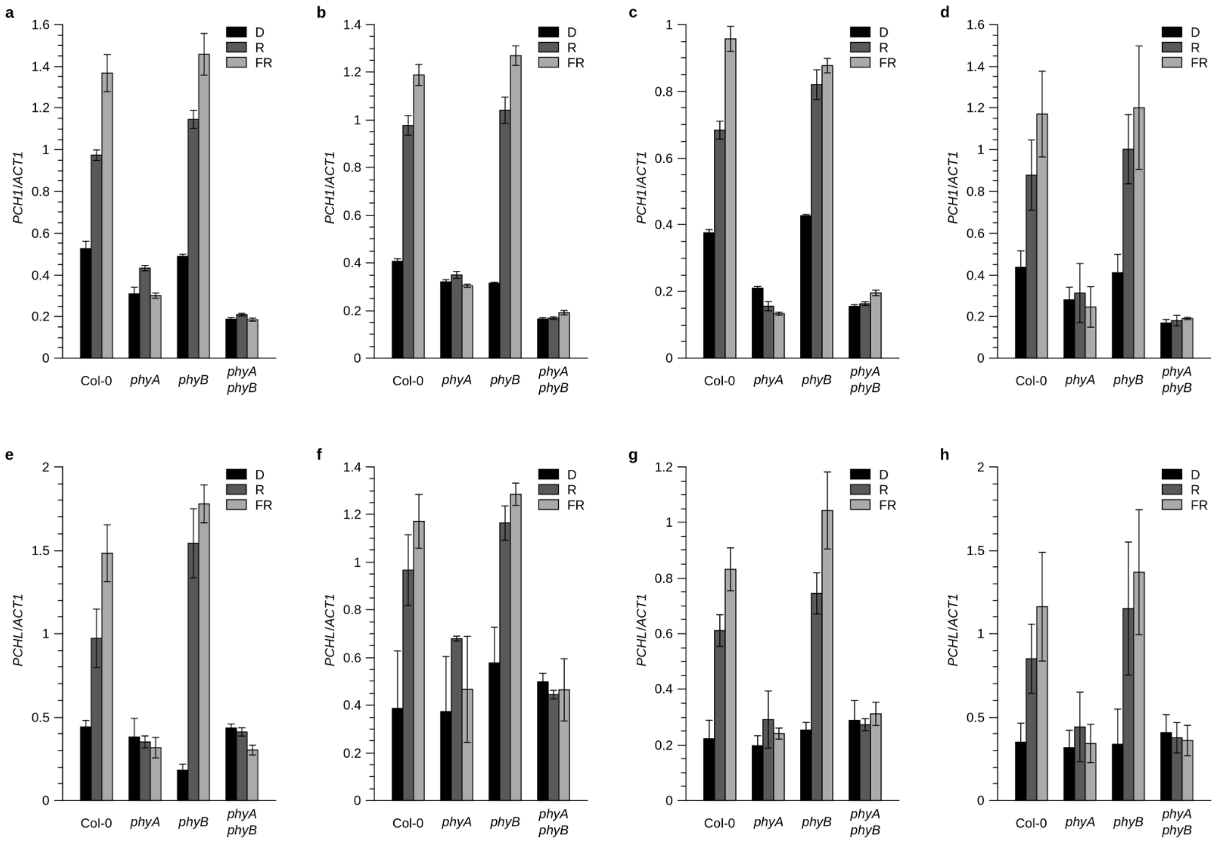

Supplementary Figure 11. Regulation of *PCH1* and *PCHL* expression by red and far-red light requires phyA. a–h Four day-old dark-grown wild type (Col-0), *phyA-211*, *phyB-9*, and *phyA-211 phyB-9* seedlings were either kept in darkness (D) or exposed to red (R, 7.5 µmol m^{-2} s^{-1}) or far-red light (FR, 6.7 µmol m^{-2} s^{-1}) for 1 h. Total RNA was extracted and qRT-PCR was performed using probes specific for either *PCH1* or *PCHL*. *ACT1* was used as internal control and expression of *PCH1* and *PCHL* is shown relative to expression of *ACT1*. Three biological replicates are shown for *PCH1* (**a–c**) and *PCHL* (**e–g**). Data of each biological replicate are means (±s.d.) of three technical replicates. **d, h** Bars show means (±s.d.) of the biological replicates. **d** and **h** are also shown in Fig. 4c and 4d.

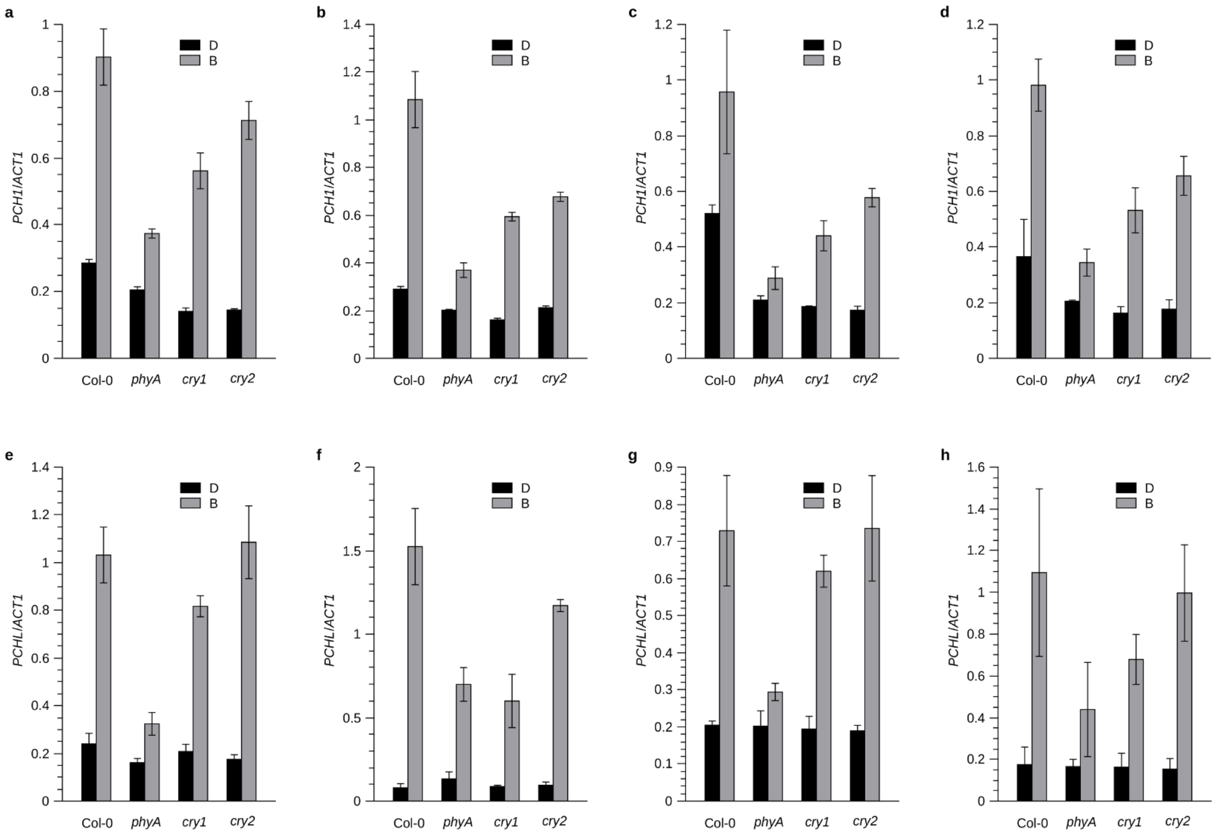

Supplementary Figure 12. PhyA regulates expression of *PCH1* and *PCHL* in response to blue light. a–h Four day-old dark-grown wild type (Col-0), *phyA-211*, *cry1-304*, and *cry2-1* seedlings were either kept in darkness (D) or exposed to blue light (B, 5.5 µmol m^{-2} s^{-1}) for 1 h. Total RNA was extracted and qRT-PCR was performed using probes specific for either *PCH1* or *PCHL*. *ACT1* was used as internal control and expression of *PCH1* and *PCHL* is shown relative to expression of *ACT1*. Three biological replicates are shown for *PCH1* (**a–c**) and *PCHL* (**e–g**). Data of each biological replicate are means (±s.d.) of three technical replicates. **d, h** Bars show means (±s.d.) of the biological replicates.

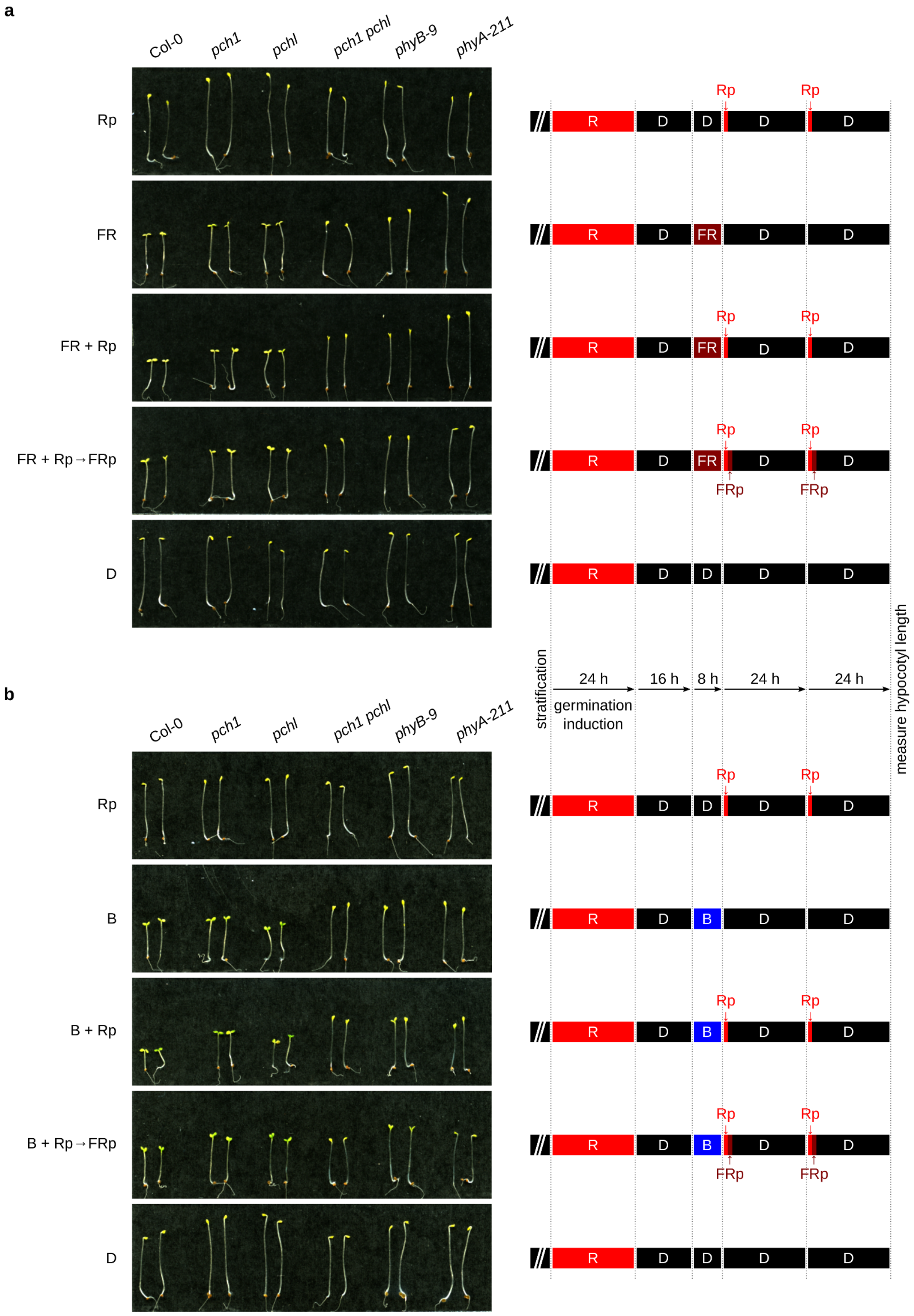

Supplementary Figure 13. PCH1 and PCHL are required for phyB responsiveness amplification by FR and B. a, b Dark-grown wild type (Col-0), *pch1*, *pchl*, *pch1 pchl*, *phyA-211*, and *phyB*-9 seedlings were pre-treated with either FR (25 µmol m^{-2} s^{-1}) (**a**) or B (25 µmol m^{-2} s^{-1}) (**b**) and given a R pulse (5 min, 50 µmol m^{-2} s^{-1}), either followed by a FR pulse (5 min, 50 µmol m^{-2} s^{-1}) or not; the light pulses were repeated after one day. See Fig. 4e and 4f for quantification of hypocotyl growth. A schematic drawing illustrating the light treatments is shown on the right.

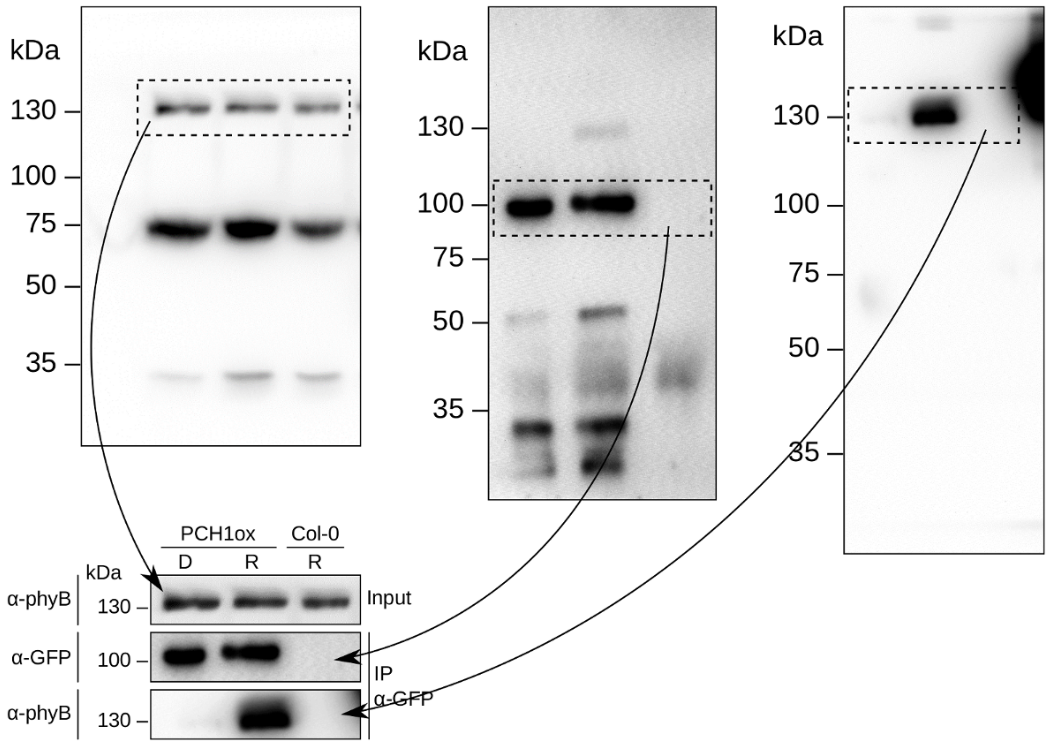

Supplementary Figure 14. Uncropped Fig. 1c.

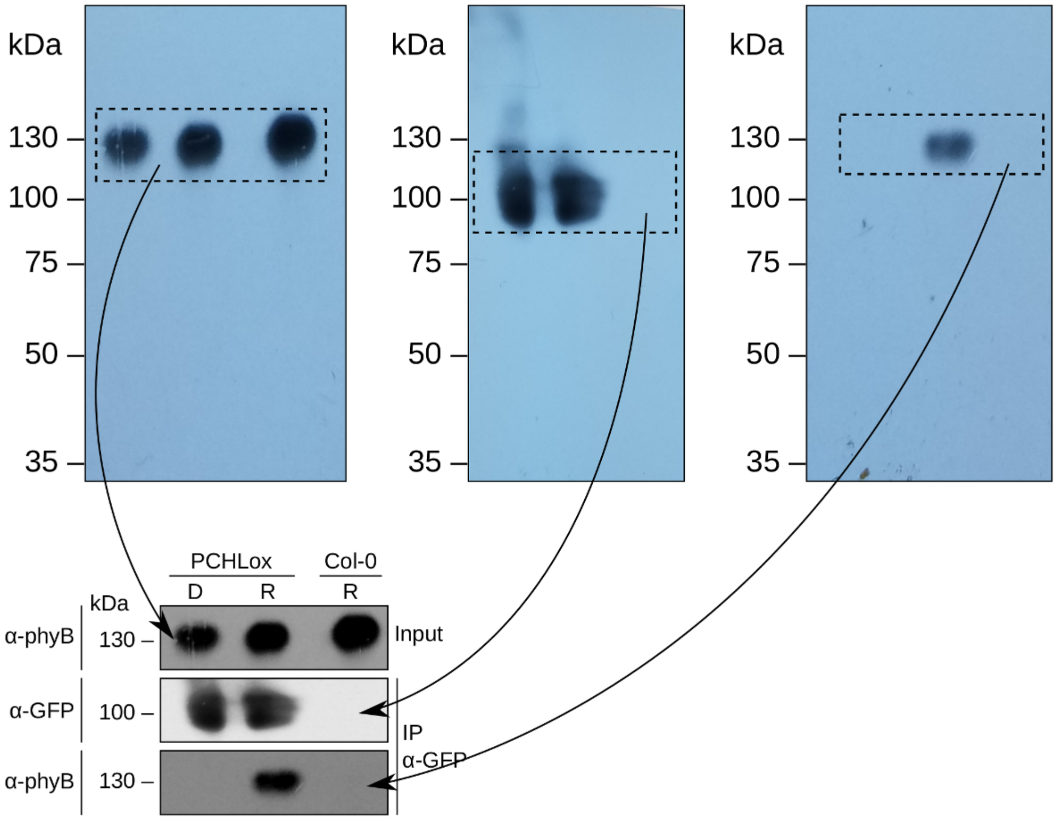

Supplementary Figure 15. Uncropped Fig. 1d.

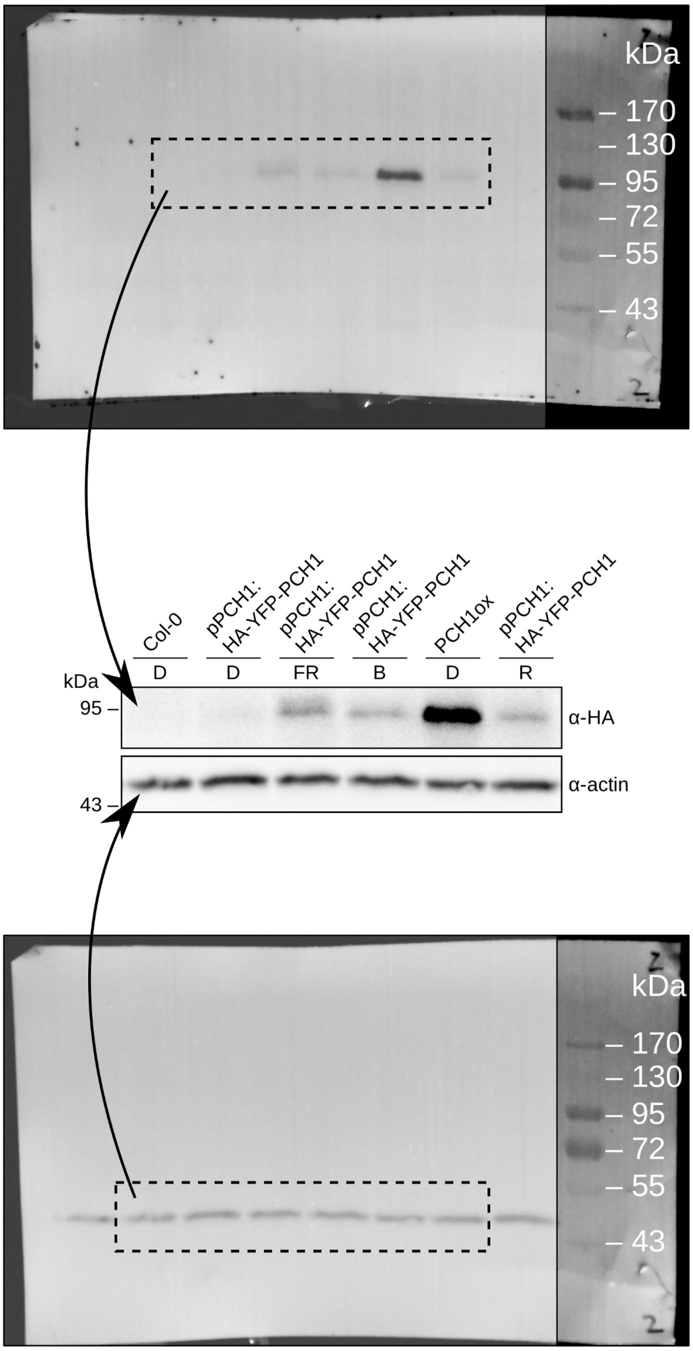

Supplementary Figure 16. Uncropped Fig. 4a.

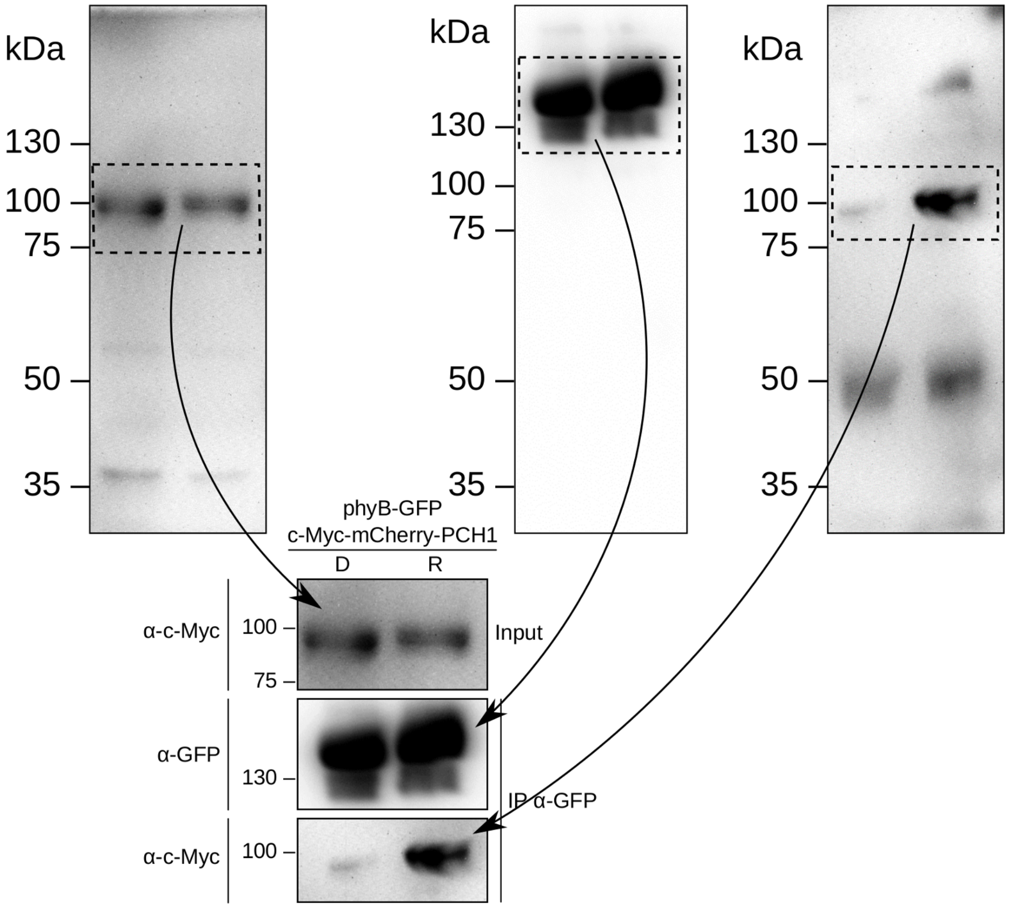

Supplementary Figure 17. Uncropped Supplementary Fig. 1b.

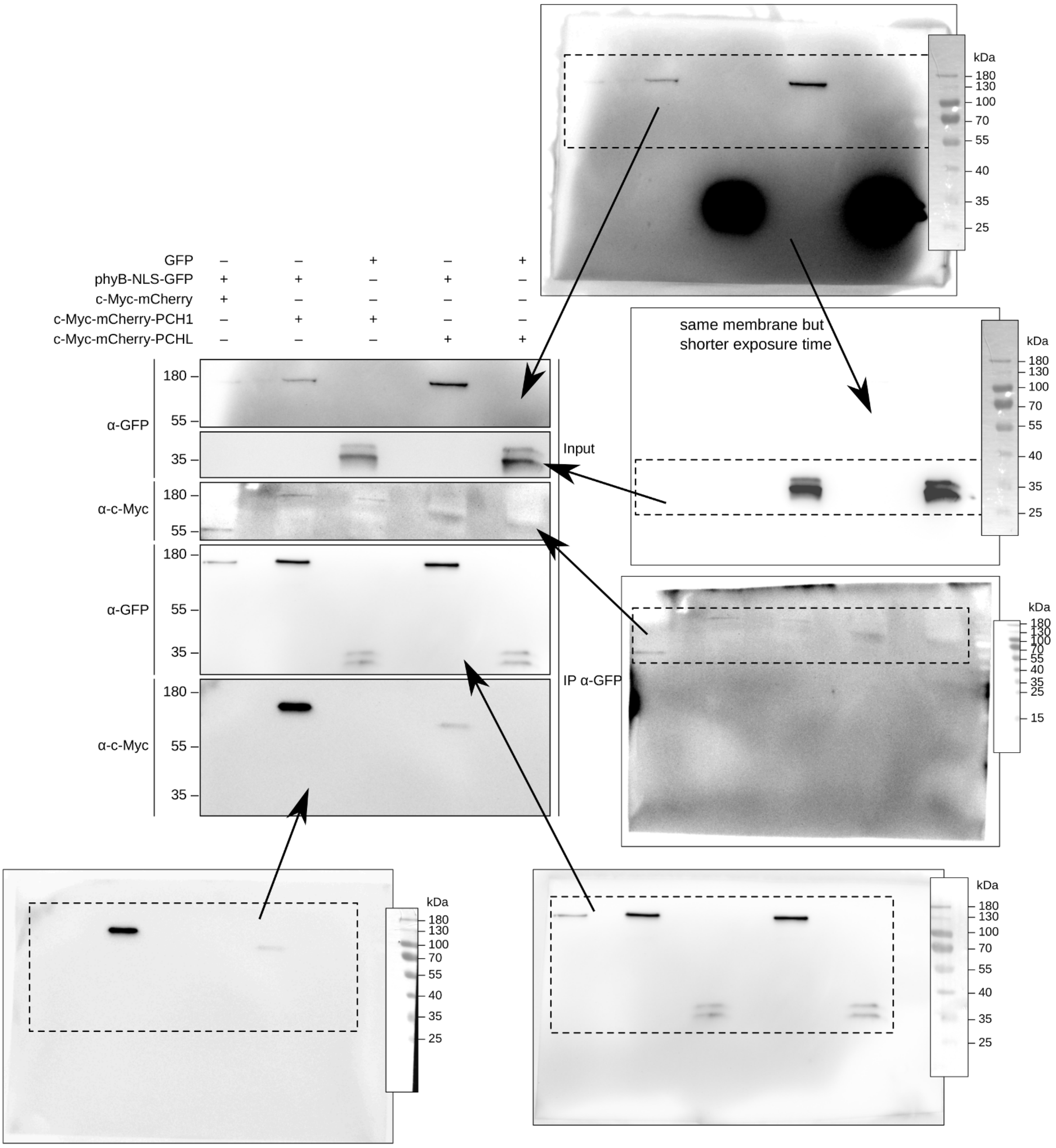

Supplementary Figure 18. Uncropped Supplementary Fig. 1c.

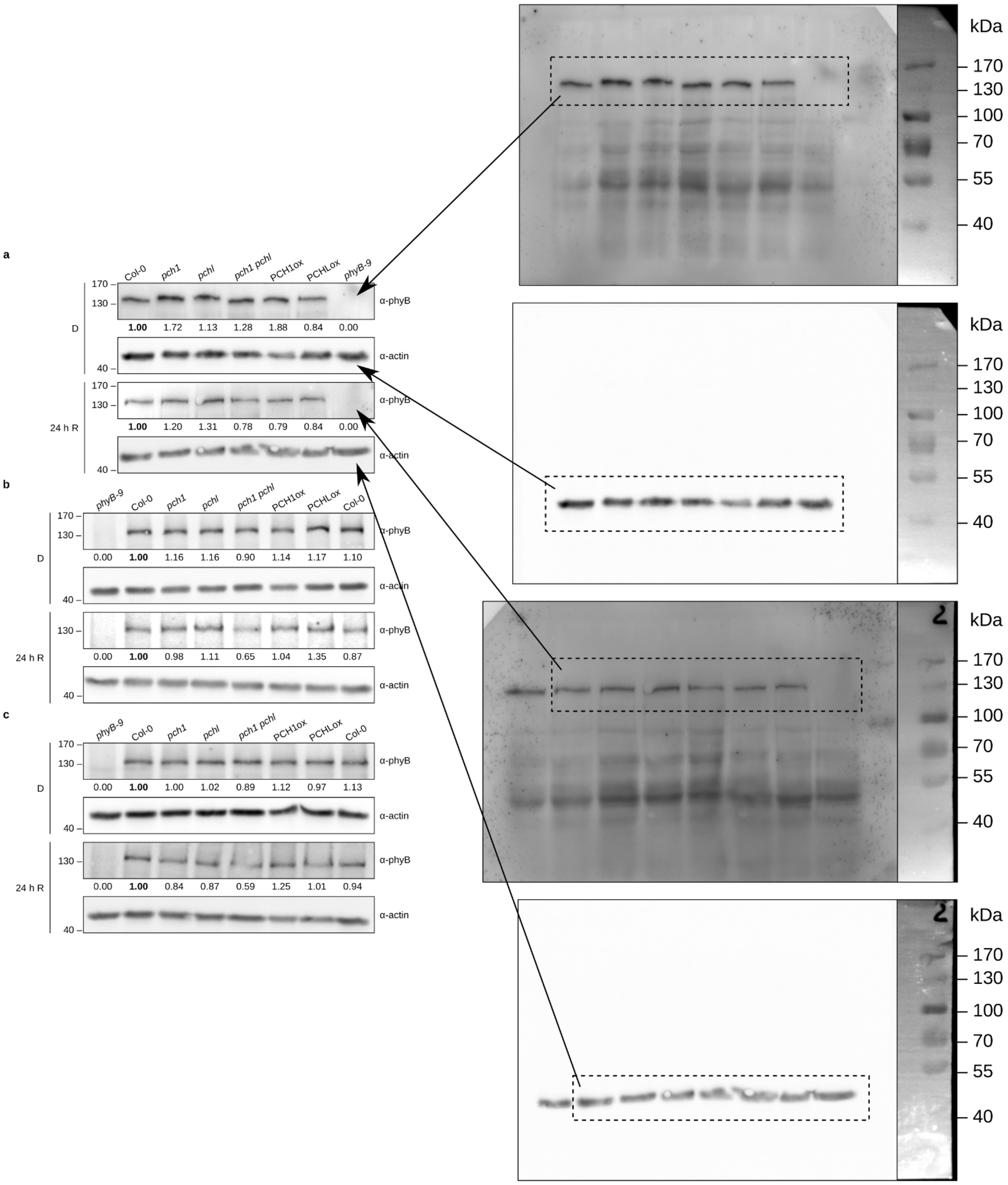

Supplementary Figure 19. Uncropped Supplementary Fig. 5a.

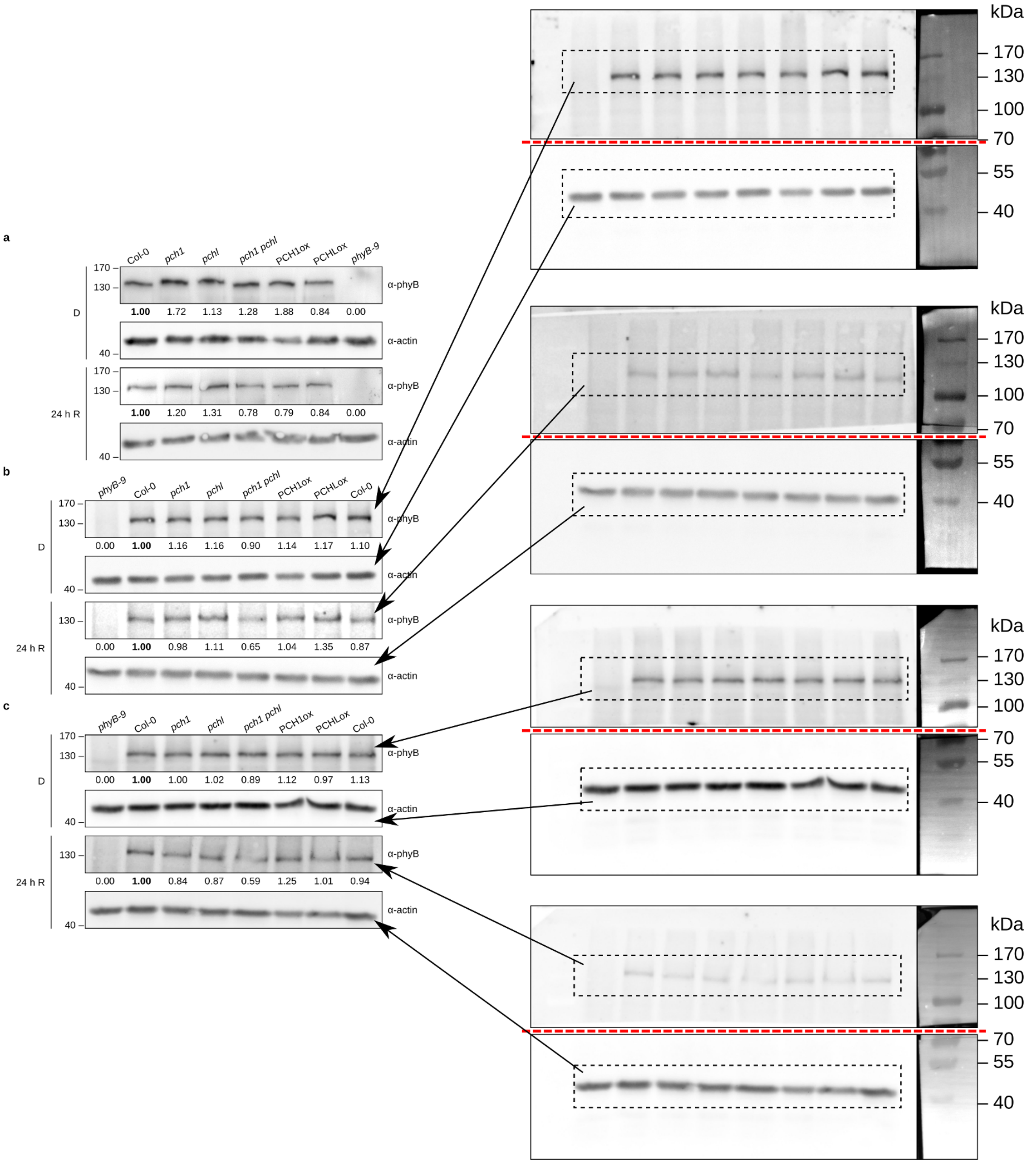

Supplementary Figure 20. Uncropped Supplementary Fig. 5b and 5c.

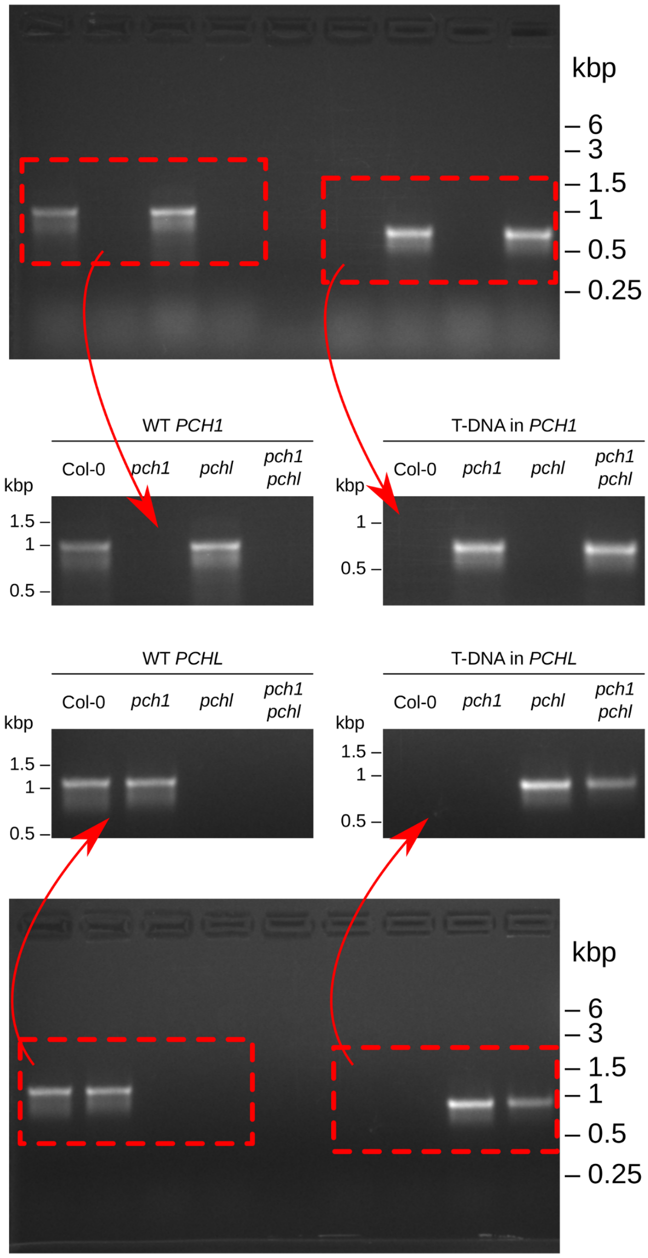

Supplementary Figure 21. Uncropped Supplementary Fig. 6a.

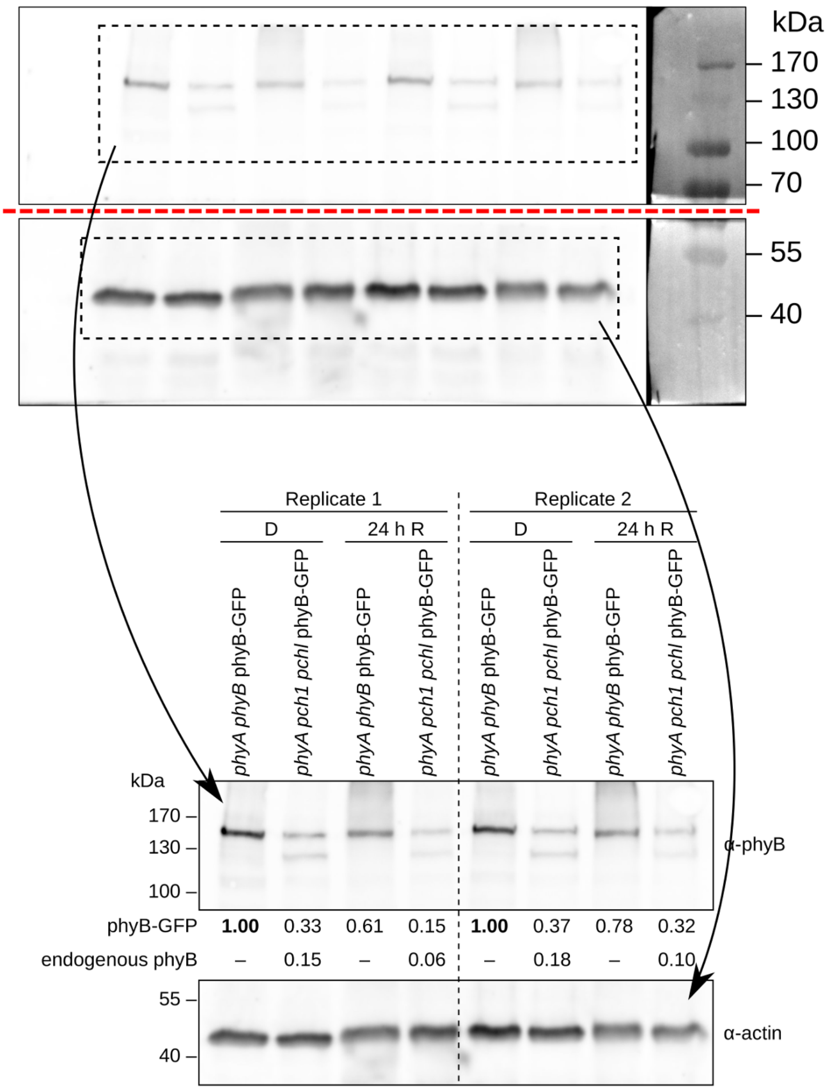

Supplementary Figure 22. Uncropped Supplementary Fig. 8.

SUPPLEMENTARY NOTE 1

Vectors and cloning

For yeast two-hybrid experiments, pGBK-MCS [4] was used as the GAL4-DNA binding domain vector, in conjunction with the modified C-terminal GAL4 activation domain vector pCGADT7ah [5], and pCGADT7ah-PHYB [4]. *PCH1* (cDNA, At2g16365.2) was amplified using primers DS178/DS179, cut with *Bcl*I/*Spe*I and cloned into the *BamH*I/*Spe*I sites of pGBK-MCS to create pGBK-PCH1 (pBE76). *PCHL* (cDNA, At4g34550) was amplified using primers BE061/BE062, cut with *BamH*I and cloned into the *BamH*I site of pGBK-MCS to create pGBK-PCHL (pBE32)

For expression of phyB and PCH1/PCHL in mammalian cells, the expression vector pcDNA3.1 (Thermo Fisher Scientific) was used. The *PHYB* CDS was amplified using primers oNK1/oPS239, and monomeric *GFP* (mGFP) [6] using oPS119/oPS238, both fragments were then cloned into *Acc65*I/*BamH*I cut pcDNA3.1 by Gibson assembly [7] to create pcDNA-PHYB-NLS-GFP (pPS136). The GFP control plasmid, pcDNA-GFP (pPS104), was generated by insertion of an mGFP PCR fragment (primers oPS132/oPS133) into *Xba*I/*Nhe*I cut pcDNA3.1 by Gibson assembly [7]. To generate an mCherry fusion construct, c-Myc-tagged mCherry was amplified from pCHF150myc [8] using DS465/DS466, and inserted into *Sac*I/*Xba*I cut pcDNA3.1 by Gibson assembly [7]. This vector was subsequently cut with *Sac*I, and the annealed oligonucleotides DS475/DS476 ligated in to create pcDNA-mCherry, possessing both an N-terminal dual FLAG-c-Myc tag, and a C-terminal multiple cloning site (pcDNA-FLAG-c-Myc-mCherry-MCS). PCH1 was amplified using DS178/BE069, cut with *Bcl*I/*Spe*I, and cloned into *BamH*I/*Xba*I cut pcDNA-mCherry to create pcDNA-mCherry-PCH1 (pBE114). Likewise, PCHL was amplified using BE060/BE061, cut with *BamH*I/*Xba*I, and cloned into *BamH*I/*Xba*I cut pcDNA-mCherry creating pcDNA-mCherry-PCHL (pBE115).

To generate over-expression vectors for *Arabidopsis* transformation, pPPO70v1HA [5], and pCHF150myc [8] were used. These vectors encode CaMV 35S promoter driven N-terminal HA-YFP and c-Myc-mCherry cassettes respectively. *PCH1* CDS was amplified and cut as above for pGBK-PCH1, and ligated into *BamH*I/*Xba*I cut pPPO70v1HA to generate pPPO70HA-PCH1 (pDS366), and pCHF150myc to create pCHF150myc-PCH1 (pBE50). For PCHL, the HA-YFP fusion was first amplified using ah094/ah767, cut with *Bgl*II/*Spe*I, and ligated into the *BamH*I/*Xba*I sites of pCHF5 [9] to generate pCHF70HA, which has an alternate herbicide resistance marker for Basta. *PCHL* CDS was then amplified and cut as above for pGBK-PCHL, and cloned into the *BamH*I/*Xba*I sites of pCHF70HA to create pCHF70HA-PCHL (pBE52c), and into pCHF150myc to generate pCHF150myc-PCHL (pBE060).

For expression of PCH1 from its native promoter, a promoterless vector pPPOv3 (pDS75) was first generated. pCHF5v1 [4] was cut with *Hind*III/*Xma*I, treated with Klenow fragment, and re-ligated to remove additional sequence between the terminator and Basta resistance marker. Oligos DS342/DS341/DS331/DS343 were used to amplify and remove an *Nhe*I site present within the replication origin by overlap-extension PCR, cut and ligated into flanking *Nde*I sites. Subsequently the CaMV 35S promoter was replaced with annealed oligonucleotides DS356/DS357 ligated into the *Nco*I/*Sal*I sites, generating a new multiple cloning site. Finally DS332/DS340/DS333/DS330 were used to amplify the Butafenacil resistance marker from pPPO70v1HA [4], removing a *Nco*I site, by overlap extension PCR, cut with *Sal*I/*Xho*I, and cloned into the new *Sal*I/*Xho*I sites to generate pPPOv3. The upstream region of the PCH1 promoter (1455 nt upstream of ATG start codon) was amplified using primers BE006/BE007, cut with *Nco*I/*Xma*I, and cloned into the *Nco*I/*Xma*I sites of pPPOv3. Subsequently, the HA-YFP region was cut from pPPO70v1HA with *Xma*I/*BamH*I, and ligated into the *Xma*I/*BamH*I sites. Finally *Bcl*I/*Spe*I cut PCH1 from above was cloned into the *BamH*I/*Spe*I sites to generate pPCH1-HA-YFP-PCH1 (pBE48).

For expression of mCerulean (mCer)-tagged phyB from its native promoter we first generated a T-DNA vector containing a p35S:*BamH*I-*Xba*I-mCer:terRbcS cassette. To this end, we amplified the CDS of mCer from pCHF80 [5] using the primers ah042/ah043, cut the PCR product with *BamH*I/*Nhe*I and ligated it into the *BamH*I/*Xba*I sites of pCHF5 [9], resulting in pCHF43. Then we cut pWCO35 [10] with *Pvu*II/*Pst*I and ligated the fragment containing the CDS of modified *PPO* into pCHF43 cut with *Sbf*I/*Pml*I to obtain pPPO43. pCHF43 contains a selection marker conferring resistance to Basta, while pPPO43 contains modified *PPO* that confers resistance to Butafenacil/Inspire [5]. To generate pPPO43-PHYB we cut phyB from pCHF40-PHYB [4] using *Xba*I and ligated it into pPPO43 cut with *Xba*I. Finally, we amplified a fragment containing the *PHYB* promoter (1825 nt upstream of the ATG start codon) and part of the *PHYB* CDS from *Arabidopsis thaliana* (L*er*-0 ecotype) genomic DNA using the primers ah944/ah945, cut the fragment with *Pme*I/*BamH*I and ligated it into pPPO43-PHYB digested with *Pme*I/*BamH*I, resulting in pPPO43B-PHYB (pPHYB:PHYB-mCer:terRbcS).

For purification of phyB complexes used for MS/MS analyses, we used plants expressing phyB-NLS-GFP. To obtain the T-DNA vector for transformation of *Arabidopsis* plants, we PCR amplified full-length *PHYB* with primers containing 5'-*Kpn*I/3'-*Sma*I sites and cloned it into pEZS-NL (http://deepgreen.stanford.edu). Then p35S:PHYB-GFP cassette was digested from pEZS-NL and cloned into pART27 [11]. SV40 NLS sequence was then cloned into pART27 p35S:PHYB-GFP using the *Sma*I restriction site, resulting in pART27 p35S:PHYB-NLS-GFP.

To assess the spatio-temporal activity of the *PCH1* and *PCHL* promoters i*n planta*, pPCH1:GUS and pPCHL:GUS plant vectors were generated. First, we amplified the *GUS* CDS using primers ah1159/ah1160, cut the PCR fragment with *BamH*I/*Xba*I and ligated it into the *BamH*I/*Spe*I sites of pPPOv3. Second, we amplified promoter fragments of *PCH1* (1455 nt upstream of the start ATG codon) and *PCHL* (1268 nt upstream of the start ATG

codon) using the primers BE006/BE007 and BE112/BE113, respectively, cut the PCR fragments with *Nco*I/*Xma*I and inserted them into the the *Nco*I/*Xma*I sites of pPPO3v1 containing the *GUS* CDS. Finally, the promoter-GUS fragments (pPCH1-GUS and pPCHL-GUS) were cut from these vectors using *Nco*I/*Sal*I and ligated into the *Nco*I/*Sal*I sites of pCHF5 [9] resulting in the two plasmid pBE26new and pBE75.

All plasmid constructs used in the study were verified by DNA sequencing.

Primer list

ah042: 5'-CGCGGATCCCGCTCTAGAATGGTGAGCAAGGGCGAGG-3'
ah043: 5'-CGGGGTACCGCTAGCTTAACTAGTCTTGTACAGCTCGTCCATG-3'
ah094: 5'-GGACTAGTTATCTAGAGCCCTAGGATCCGCCTTGTACAGCTCGTCCATG-3'
ah767: 5'-GAAGATCTAAAAATGGCCTACCCATACGACGTACCAGATTACG-3'
ah944: 5'-TGATAGTTTAAACATTAGTATGGGAGGGCTTAG-3'
ah945: 5'-CAATAGAAAGCGCAGGATCT-3'
ah1159: 5'-CGCGGATCCATGTTACGTCCTGTAGAAACCC-3'
ah1160: 5'-GCTCTAGATTAAAGATCTGCTTGTTTGCCTCCCTGCTG-3'
BE006: 5'-ACGTCCCATGGTATAAACTTTATTGTATAGGTTAG-3'
BE007: 5'-ACGTCCCCGGGTTGTACGTACCTACAGATCTTC-3'
BE060: 5'-GGATCCGATGTCTGAACATTTTATGGG-3'
BE061: 5'-CGGATCCTCTAGATCAACTTAAATCCTCAGCATTC-3'
BE062: 5'-GGATCCGGATGTCTGAACATTTTATGGG-3'
BE069: 5'-ACGTCACTAGTCTACCTCAAATCCCTTGCATTCC-3'
BE078: 5'-AAAATGCCAAGGAGATTCACC-3'
BE079: 5'-CAGGATGGTCTCTGCTCACTC -3'
BE112: 5'-CCATGGAATTATAAACCAAACAGC-3'
BE113: 5'-CCCGGGTTGTACCTAAAAGATAT -3'
DS178: 5'-ACGTCTGATCAAGGAATGTCTGAACATGTTATGGTTTTG-3'
DS179: 5'-ACGTCACTAGTGCCCCTCAAATCCCTTGCATTCCAAA-3'
DS245: 5'-AAAGAATGTGATCCTAGTTTATC-3'
DS246: 5'-ATACCTTAGTTGATGACGAAGAA-3'
DS330: 5'-TCTCTGCGATTTGCATGGAGTTATC-3'
DS331: 5'-CCAATCAGCTGGCTCGATCTC-3'
DS332: 5'-ACGTCGTCGACCTGCAGAGCTTTC-3'
DS333: 5'-ACGTCCTCGAGTACCCACTGGATTTTGGTTTTAG-3'
DS340: 5'-GATAACTCCATGCAAATCGCAGAGA-3'

DS341: 5’-GAGATCGAGCCAGCTGATTGG-3’
DS342: 5’-ACGTCCATATGGTGCACTCTCAGTACA-3’
DS343: 5’-ACGTCCATATGGGCCACCGCCGA-3’
DS356: 5’-CATGGTACCCGGGGCGGATCCAAAAACTAGTTAAG-3’
DS357: 5’-TCGACTTAACTAGTTTTTGGATCCGCCCCGGGTAC-3’
DS465: 5’-TACGGTGGGAGGTCTATATAAGCAGAGCTCGCCGCCACCATGGCCGAGGAGCAGAAG-3’
DS466: 5’-GCTGATCAGCGGGTTTAAACGGGCCCTTCATCTAGACCCGGGATCCGCCTTGTACAGCTC-3’
DS475: 5’-CGTTTAGTGAACCGTCAGATCCGCCACCATGGTAGACTACAAAGACGATGACGACAAGCT-3’
DS476: 5’-TGTCGTCATCGTCTTTGTAGTCTACCATGGTGGCGGATCTGACGGTTCACTAAACGAGCT-3’
LBb1.3: 5’-ATTTTGCCGATTTCGGAAC-3’
oNK1: 5’-CCAAGCTGGCTAGCGTTTAAACTTAAGCTTGGTACCATGGTTTCCGGAGTCGGGGGTAG-3’
oPS119: 5’-CAGAATTCCACCACACTGGACTAGTGGATCTTACTTGTACAGCTCGTCCATGCCGAG-3’
oPS132: 5’-CGACTCACTATAGGGAGACCCAAGCTGGATGTACCCATACGACGTACCAGATTACGCTGCTAGCTACCCATACGACGTACCAGATTACGCTGCTAGCATGGTGAGCAAGGGCGAGGAGC-3’
oPS133: 5’-CTGATCAGCGGGTTTAAACGGGCCCTCTAGCTAGTTATCTAGAGCCCTAGGATCCGCGCTGCCTCCGCTGCCACCCTTGTACAGCTCGTCCATGCCGAG-3’
oPS238: 5’-CCAAAAAAGAAGAGAAAGGTAGAAGACCCCGGAACTAGTGGTGGCAGCGGA-3’
oPS239: 5’-GGGGTCTTCTACCTTTCTCTTCTTTTTTGGATATGGCATCATCAGCATCATGTCACCAC-3’

Primers and probes for qRT-PCR analyses

PCH1 forward primer: 5’-AATCATCGGCAGAGACTAATACCTTAG-3’
PCH1 reverse primer: 5’-GCCTTTTATTCCTTTTCCCTTAGTTG-3’
PCH1 probe: FAM-5’-TACATCTTTCCGGCTCCATTTCTTCGTCA-3’-ZEN/BFQ
PCHL forwad primer: 5’-GCGTATATGGTCGGACTCTGAAA-3’
PCHL reverse primer: 5’-TCTTCGAAAACCATCATGTTCTTG-3’
PCHL probe: FAM-5’-CACAAGTCAAACGTCTGAAGACAAACGCA-3’-ZEN/BFQ
ACT1 forward primer: 5’-GGCTCCAAGCAGCATGAAG-3’
ACT1 reverse primer: 5’-ACCCTCCAATCCAGACAGAGTATT-3’
ACT1 probe: JOE-5’-CAAAGTCGTTGCCCCTCCAGAGAGG-3’-BHQ1

SUPPLEMENTARY REFERENCES

1. Larkin, M. A. *et al.* Clustal W and Clustal X version 2.0. *Bioinformatics* **23,** 2947–2948 (2007).
2. Waterhouse, A. M., Procter, J. B., Martin, D. M. A., Clamp, M. & Barton, G. J. Jalview Version 2 – a multiple sequence alignment editor and analysis workbench. *Bioinformatics* **25,** 1189–1191 (2009).
3. Huang, H. *et al.* PCH1 integrates circadian and light-signaling pathways to control photoperiod-responsive growth in *Arabidopsis*. *eLife* **5,** 1–27 (2016).
4. Sheerin, D. J. *et al.* Light-activated phytochrome A and B interact with members of the SPA family to promote photomorphogenesis in Arabidopsis by reorganizing the COP1/SPA complex. *Plant Cell* **27,** 189–201 (2015).
5. Rausenberger, J. *et al.* Photoconversion and nuclear trafficking cycles determine phytochrome A's response profile to far-red light. *Cell* **146,** 813–825 (2011).
6. von Stetten, D., Noirclerc-Savoye, M., Goedhart, J., Gadella, T. W. J. & Royant, A. Structure of a fluorescent protein from *Aequorea victoria* bearing the obligate-monomer mutation A206K. *Acta Crystallogr Sect F Struct Biol Cryst Commun* **68,** 878–882 (2012).
7. Gibson, D. G. *et al.* Enzymatic assembly of DNA molecules up to several hundred kilobases. *Nat Methods* **6,** 343–345 (2009).
8. Possart, A. & Hiltbrunner, A. An evolutionarily conserved signaling mechanism mediates far-red light responses in land plants. *Plant Cell* **25,** 102–114 (2013).
9. Hiltbrunner, A. *et al.* Nuclear accumulation of the phytochrome A photoreceptor requires FHY1. *Curr Biol* **15,** 2125–2130 (2005).
10. Hanin, M. *et al.* Gene targeting in *Arabidopsis*. *Plant J* **28,** 671–677 (2001).
11. Gleave, A. P. A versatile binary vector system with a T-DNA organisational structure conducive to efficient integration of cloned DNA into the plant genome. *Plant Mol Biol* **20,** 1203–1207 (1992).
